# Coronary angioplasty in octogenarians with emergent coronary syndromes: study protocol for a randomized controlled trial

**DOI:** 10.1186/1745-6215-15-349

**Published:** 2014-09-04

**Authors:** Berglind Libungan, Geir Hirlekar, Per Albertsson

**Affiliations:** Department of Cardiology, Sahlgrenska University Hospital, Blå stråket 3, vån 1, SE-413 45 Gothenburg, Sweden

**Keywords:** Elderly, Myocardial infarction, Conservative therapy, Invasive therapy, Acute coronary syndrome

## Abstract

**Background:**

Invasive treatment (coronary angiography and intervention if feasible) of patients with acute coronary syndrome (ACS) has been shown to lead to better outcomes than medical therapy alone, but the elderly have been under-represented in many of the studies. In the elderly, medical therapy is common in ACS. Fear of complications related to the procedure and unclear benefit in older patients are common reasons for invasive procedures being withheld. Our hypothesis is that invasive treatment of elderly patients with ACS will lead to a better outcome in terms of survival and quality of life than medical therapy alone, with acceptable risk.

**Methods/Design:**

This multicenter, randomized controlled trial of patients 80 years of age and over has two parallel treatment arms, a medical group and an invasive group. In Swedish hospitals, 200 patients with non-ST elevation myocardial infarction or unstable angina will be randomized to medical or invasive treatment strategy. The primary outcome measure is the combined endpoint major adverse cardiac or cerebrovascular event (MACCE) within one year. Secondary outcome measures include quality of life, angina, and adverse events such as bleeding. Assessments will be conducted during hospitalization, at 1 month after allocation, and at 12 months.

**Discussion:**

This study seeks to determine the efficacy and safety of invasive and medical treatment strategies in the elderly with ACS. The study is currently recruiting.

**Trial registration:**

ClinicalTrials.gov trial identifier: NCT02126202. Registered on 7 January 2014.

## Background

In acute coronary syndrome (ACS), large randomized studies have shown a survival benefit of invasive treatment strategy over medical therapy [1, 2]. Under-representation in clinical trials and possibly fear of complications in older patients has led to a suspected under-utilization of invasive treatment in the elderly compared to that in younger population groups. However, a substudy from a large randomized trial [3] showed that the oldest patients appeared to benefit the most. In 2007, the American Heart Association published a statement [4] in order to highlight the problem. In contrast, a recent trial [5] failed to confirm survival benefit in elderly patients with non-ST elevation myocardial infarction ACS unless cardiac enzyme levels were substantially elevated.

### Study objectives and hypothesis

This randomized controlled trial (RCT) aims to address a fundamental question: which treatment strategy in elderly patients with myocardial infarction results in a better outcome with acceptable risk? Specifically, which treatment strategy improves survival and results in less morbidity and better quality of life? Equally important are the adverse events associated with each treatment strategy.

We hypothesize that revascularization in elderly patients with ACS will lead to gain in terms of outcome and quality of life compared to medical therapy alone. Secondly, we hypothesize that adverse events will occur, but will be within an acceptable range.

## Methods/Design

### Design of trial

The octogenarians study is an academically sponsored and principal investigator-initiated study. The study design adheres to the ‘CONsolidated Standards of Reporting Trials’ (CONSORT) statement [6].

The study is a phase 4 RCT with two parallel treatment arms: invasive therapy and medical therapy. Invasive therapy will include a coronary angiography and, if feasible, revascularization with percutaneous coronary intervention (PCI) or coronary artery bypass grafting (CABG). Blinding of study participants is not possible due to the invasive treatment arm, which makes the trial an open-label trial.

### Participants and recruitment

The trial is a multicenter study. Participants will be recruited from different sites in the southern and southwestern parts of Sweden. The hospitals participating are Sahlgrenska University Hospital in Gothenburg, Norra Älvborg Regional Hospital in Trollhättan, Skåne University Hospital in Lund, and Skaraborg Hospital in Skövde.

Participants will be identified by the treating physicians at these hospitals. Patients who meet the inclusion criteria will be approached by a physician who will provide a brief summary of the study. If the patient is interested in taking part, he or she will be asked to provide written informed consent. The inclusion criteria are: equal or greater than 80 years of age; ACS with ischemic symptoms (mainly chest pain) lasting over 10 minutes within the previous 72 hours, and ischemic ST-segment depression ≥ 1 mm and/or elevated troponin I, troponin T, or CK-MB; and written informed consent provided before randomization.

The exclusion criteria are: PCI within 30 days prior to randomization; suspected ongoing active internal bleeding; ST-segment elevation of ≥ 1 mm in two contiguous leads on ECG (electrocardiogram) enrollment in another study that has not completed the follow-up phase; known allergy to aspirin or P2Y_12_ antagonists; severe dementia; expected limited 1-year survival due to another disease(s); and unwillingness to participate in the trial or expected problems with compliance. Patients who meet the inclusion criteria but who are not able to participate for various reasons will be registered in a trial screening log.

### Ethics and procedure

The ethical aspects of this study have been approved by the Ethical Review Board in Gothenburg (Diarie number 157–09). The project will be conducted according to the World Medical Association Declaration of Helsinki [7].

### Randomization

The age of participants (between 80 and 84-years-old or ≥85-years-old) will be used to determine the subgroup for random allocation. This stratification is due to the possibility of unbalanced treatment arms regarding age. The random assignment to one of the two treatment arms will be conducted independently of the project staff by using sequentially numbered and sealed opaque envelopes. The envelopes will contain the group allocation on a written insert, based on a predetermined random computer-generated sequence.

### Crossover

Efforts should be made to keep the initial treatment allocation, but a certain crossover rate can be expected. Patients randomized to the invasive treatment group will receive medical treatment in case of coronary anatomy unsuitable for intervention. Patients in the medical treatment group will undergo coronary angiography with possible revascularization in case of ischemia leading to refractory chest pain, hemodynamic instability (including cardiogenic chock), heart failure, or life-threatening cardiac arrhythmias.

### Intervention procedures

*Invasive treatment (intervention arm of the RCT)*: coronary angiography will be performed with the use of 5 to 8 F catheters via the femoral or radial approach at the operator’s discretion. Revascularization with PCI or CABG will be performed if feasible and if acceptable to the patient. For all patients, standard medication (as mentioned below) will be administered if the patient has no contraindications. If PCI is performed, the patients will be treated with heparin or bivalirudin according to local guidelines and at the operator's discretion. *Medical treatment (control arm of the RCT)*: patients randomized to medical treatment will receive optimized medical treatment and will not undergo a coronary angiography.

### Trial medication

The following medication is recommended but not mandatory in both treatment arms.

Aspirin will be administered to all patients on a daily basis in a maintenance dose of between 75 and 160 mg. If the patient is previously untreated with aspirin, a loading dose of between 160 and 320 mg will be given.

Other antiplatelet medications include clopidogrel (with a loading dose of between 300 and 600 mg) or ticagrelor (loading dose of 180 mg) will be administered as soon as possible, and always before the coronary angiography, in patients randomized to invasive treatment.

Treatment with a statin (such as atorvastatin) will be started as soon as possible. Beta-blockers are often included as an anti-ischemic treatment in the absence of contraindications. Long-term treatment is recommended in the case of reduced left ventricular function. Nitroglycerin (intravenous, oral, sublingual or buccal) is recommended for symptom relief if necessary. ACE inhibitors are indicated long-term in all patients with heart failure (left ventricular ejection fraction ≤40%) and in patients with diabetes mellitus or hypertension.

### Definitions

Blood samples for hemoglobin will be obtained at baseline, and if there is suspicion of bleeding. Blood samples for platelet count will be obtained at baseline and if there is suspicion of bleeding. Blood samples for potassium, sodium, and creatinine will be obtained at baseline. Blood samples for CK-MB (creatine kinase-MB) and/or Troponin Tor Troponin I will be obtained at baseline, and if there are symptoms of myocardial ischemia. All-cause mortality will be analyzed for this study. For the purposes of this study ST-elevation myocardial infarction (STEMI) will be defined as chest pain lasting more than 10 minutes in combination with ST-segment elevation (more than 2 mm in two anterior leads, or more than 1 mm in two inferior leads), or a newly developed left bundle branch block and elevated cardiac enzymes/biochemical markers, or a new Q-wave in two continuous leads on ECG. Non-ST-elevation myocardial infarction (NSTEMI) will be defined as chest pain lasting more than 10 minutes in combination with elevated cardiac troponin levels. Peri- and post-myocardial infarction will be defined as a myocardial infarction developing within 24 hours of PCI. This includes STEMI and NSTEMI with elevated biochemical markers more than three times normal level unit. A recurrent intervention is considered to be all unplanned revascularizations after the index procedure (CABG or PCI). All non-scheduled hospitalizations due to chest pain, arrhythmias not experienced previously, or congestive heart failure not experienced previously will be considered to be recurrent hospitalization for cardiac reasons. Stroke will be defined as all ischemic attacks resulting in neurological disorders, including transitory ischemic attack (TIA).

### Outcome and safety measurements

There are several secondary outcome measures for this trial. The definitions of some of the outcome measures are. Firstly, the grade of angina pectoris as determined by the patient’s perception of angina pectoris according to the Seattle Angina Questionnaire (SAQ) [8]. This assessment will be optional for the patient. Secondly, the patient’s perception of quality of life according to SF-36 [9]. This assessment will also be optional for the patient. Thirdly, the physician’s perception of frailty at enrollment, according to the Canadian Study of Health and Aging (CSHA) Clinical Frailty Scale [10]. Fourthly, major bleeding, defined as either intracranial bleeding, a decrease in hemoglobin level of more than 50 g/L, or bleeding requiring surgery. Fifthly, minor bleeding, defined as a decrease in hemoglobin level of more than 30 g/L (but less than 50 g/L), spontaneous gross hematuria, hematemesis, hematoma, or pseudoaneurysm requiring treatment (other than surgery). A timeline of the interventions and measurements is shown in Table 1.

**Table 1:** **Schedule of enrollment, interventions, assessments, and outcome/safety measures for the octogenarian study**

	STUDY PERIOD						
	Enrollment	allocation	Post-allocation				Close-out
Time point**		**0**	**day 1**	**in-hospital**	**at discharge**	**day 30**	**1 year**
**Enrollment:**							
Eligibility screen	X						
Informed consent	X						
History and ECG	X						
Allocation		X					
**Interventions:**							
Medical treatment*			X				
Invasive treatment strategy				X			
**Assessments:**							
Echocardiography and frailty score	X						
QoL and angina questionnaire	X						X
Adverse events				X	X	X	X
If symptoms, additional assessments**					X	X	X
**Outcome:**							
MACCE***						X	X^§^
Myocardial infarction							X
All-cause mortality							X
**Safety measure: bleeding**				X	X	X	X

*Medical treatment will be given to all study participants regardless of treatment allocation, provided that there are no contraindications.

**If symptoms of ischemia: ECG, CK-MB, and cardiac troponins. If symptoms of bleeding: hemoglobin and platelet count.

***Combined endpoint: MACCE: major adverse cardiac or cerebral event.

^§^Primary endpoint of the study.

QoL: Quality of life, ECG: Electrocardiogram.

### Reporting of adverse events

According to Swedish legislation, the investigator is responsible for reporting of safety information to local authorities. An adverse event (AE) is any unfavorable sign or symptom associated with the study agents or the coronary intervention. A serious adverse event (SAE) is defined as any AE occurring that results in: death, a life-threatening event, the prolongation of existing hospitalization or new unplanned hospitalization, persistent or significant disability, or intracranial hemorrhage.

### Outcome

#### Primary outcome

The primary outcome measure is the combined endpoint of a major adverse cardiac or cerebrovascular event (MACCE) within 1 year. Assessments will be conducted during hospitalization, discharge, after 30 days, and after 1 year, as shown in Table 1.

### Secondary outcome

Secondary outcome measures include: any MACCE within 1 month, death at 1 year, any myocardial infarction at 1 year, death or myocardial infarction at 1 year, major bleeding at 30 days, and minor bleeding at 30 days.

### Sample size

According to previous trials, such as the TACTICS-TIMI 18 elderly substudy [3], we expect a MACCE rate of 40% within one year of allocation with the conservative strategy, and that this could be reduced to 20% with an invasive strategy. With 80% power and an α level of <0.05, this trial will require 82 individuals in each group. Due to possible dropout, 200 patients will be included in the trial; 100 in each treatment arm. No interim analysis is planned.

### Data and statistical analysis

Data will be entered into each patient’s case report form (CRF). The principal investigator is the data custodian. Data will be screened and, if necessary, cleaned (the process of detecting and correcting corrupt or inaccurate records from a record set, table, or database) in order to ensure validity and integrity. The statistical analysis will be managed according to the intention-to-treat principle.

## Discussion

This study seeks to determine the efficacy and safety of invasive and medical treatment strategies in elderly patients with ACS. In doing so, the study will advance our understanding of therapy choice in elderly patients with ACS. The different treatment strategies have the potential to reduce angina pectoris and complications, and improve quality of life and survival.

## Trial status

Recruitment to the study commenced in October 2009 and is still continuing to recruit patients. The recruitment of all patients will hopefully be finished in year 2015.

## Abbreviations

ACS: Acute coronary syndrome
MI: Myocardial infarction
PCI: Percutaneous coronary intervention
TnT: Troponin T
TnI: Troponin I
QoL: Quality of life
ECG: electrocardiogram.
